# Arctigenin Inhibits Liver Cancer Tumorigenesis by Inhibiting Gankyrin Expression via C/EBPα and PPARα

**DOI:** 10.3389/fphar.2018.00268

**Published:** 2018-03-27

**Authors:** Ying Sun, Yu-jun Tan, Zhan-zhao Lu, Bing-bing Li, Cheng-hong Sun, Tao Li, Li-li Zhao, Zhong Liu, Gui-min Zhang, Jing-chun Yao, Jie Li

**Affiliations:** ^1^Shandong New Time Pharmaceutical Co., Ltd., Lunan Pharmaceutical Group Co., Ltd., Linyi, China; ^2^State Key Laboratory of Generic Manufacture Technology of Chinese Traditional Medicine, Lunan Pharmaceutical Group Co., Ltd., Linyi, China; ^3^Center for New Drug Safety Evaluation of Lunan Pharmaceutical, Lunan Pharmaceutical Group Co., Ltd., Linyi, China

**Keywords:** arctigenin, C/EBPα, PPARα, gankyrin, hepatocellular carcinoma

## Abstract

Burdock (*Arctium lappa*) is a popular vegetable in China and Japan that is consumed for its general health benefits. The principal active component of burdock is arctigenin, which shows a range of bioactivities *in vivo* and *in vitro*. Here, we investigated the potential anti-tumor effects of arctigenin using two human hepatocellular carcinoma (HCC) cell lines, HepG2 and Hep3B, and sought to elucidate its potential mechanisms of action. Our results showed that arctigenin treatment inhibited cell growth in both HepG2 and Hep3B cell lines (IC_50_ of 4.74 nM for HepG2 cells, and of 59.27 nM for Hep3B cells). In addition, migration, invasion, and colony formation by HepG2 cells were significantly inhibited by arctigenin. By contrast, treatment of Hep3B cells with arctigenin did not alter these parameters. Arctigenin also significantly reduced the levels of gankyrin mRNA and protein in HepG2 cells, but not in Hep3B cells. A luciferase assay indicated that arctigenin targeted the -450 to -400 region of the gankyrin promoter. This region is also the potential binding site for both C/EBPα and PPARα, as predicted and confirmed by an online software analysis and ChIP assay. Additionally, a co-immunoprecipitation (Co-IP) assay showed that binding between C/EBPα and PPARα was increased in the presence of arctigenin. However, arctigenin did not increase the expression of C/EBPα or PPARα protein. A binding screening assay and liquid chromatography–mass spectrometry (LC–MS) were performed to identify the mechanisms by which arctigenin regulates gankyrin expression. The results suggested that arctigenin could directly increase C/EBPα binding to the gankyrin promoter (-432 to -422 region), but did not affect PPARα binding. Expression of gankyrin, *C/EBPα*, and *PPARα* were analyzed in tumor tissues of patients using real-time PCR. Both *C/EBPα* and *PPARα* showed negative correlations with gankyrin. In tumor-bearing mice, arctigenin had a significant inhibitory effect on HCC growth. In conclusion, our results suggested that arctigenin could inhibit liver cancer growth by directly recruiting C/EBPα to the gankyrin promoter. PPARα subsequently bound to C/EBPα, and both had a negative regulatory effect on gankyrin expression. This study has identified a new mechanism of action of arctigenin against liver cancer growth.

## Introduction

Plants have been a vital food source for humans since our species first evolved. In addition to their use as basic foodstuffs, plants have also been exploited for medicinal purposes in order to avoid or treat diseases and to extend lifespan. As a result, a large number of plants have been identified for use because of their multiple beneficial functions, particularly in traditional medicine. However, in recent decades there has been much dispute on the benefits of traditional Chinese medicine (TCM) compared to those of Western-based medical strategies due to the lack of clarity regarding the mechanisms of action of plant compounds in TCM (Brand and Zhao, 2017; Fleischer et al., 2017; Yang et al., 2017). To overcome this problem, modern Chinese medicine focuses on extracting and purifying the active ingredients from herbal remedies, such as from the fruit of *Forsythia suspense* Vahl (*Forsythiae Fructus*) and from *Ginkgo biloba* extracts, and to identify their potential mechanisms of action. As a consequence, TCM has been increasingly applied in the treatment of clinical diseases (Swarup et al., 2008; Wu et al., 2014; Lu G.D. et al., 2015; Wang et al., 2018).

Burdock root (*Arctium lappa* L.) has remarkable health benefits and is widely eaten as a vegetable in Asia, especially in China. In recent years, the active component of burdock root, arctigenin, has been demonstrated to possess multiple pharmacological functions and to have anti-tumor, anti-oxidant, anti-inflammatory, anti-viral, neuroprotective, and endoplasmic reticulum (ER) stress regulatory effects (Tsai et al., 2011; Gu et al., 2012; Zhang N. et al., 2013). Previous studies have demonstrated that arctigenin is a powerful antineoplastic agent that can suppress proliferation of cancer cells and promote apoptosis through various mechanisms. For example, arctigenin induces apoptosis in the human lung adenocarcinoma cell line A549, regulates the NOX1 and p-38MAPK pathways in the human breast cancer cell line MDA-MB-231, increases the Bax/Bcl2 protein ratio in the human breast cancer cell line MCF-7, regulates the NFκB, PI3K/AKT, and Stat3 pathways in the human prostate cancer cell line LNCaP, induces cell cycle arrest at the G0/G1 phase in gastric cancer cells, and regulates the Wnt/β-catanin signaling pathway to block the cell cycle at the G2/M phase in colorectal cancer cells (Jeong et al., 2011; Susanti et al., 2012; Wang et al., 2014; Su and Wink, 2015). Arctigenin has also been reported to induce apoptosis in the hepatocellular carcinoma (HCC) cell lines HepG2 and SMMC7721 by decreasing the mitochondrial outer membrane potential and enhancing Bax expression; however, arctigenin does not affect normal hepatic cells (Lu Z. et al., 2015). These studies suggest that arctigenin has an anti-tumor effect via different mechanisms in different tumor types (Liu et al., 2014; Li A. et al., 2015). However, the mechanisms of arctigenin activity in tumor resistance are not yet fully understood.

Hepatocellular carcinoma is one of the most prevalent cancers worldwide, particularly in Asian countries (Yim et al., 2015). Although several treatment strategies for HCC are available, such as surgical resection and radiation and drug treatments, successful therapy tends to be confined to early stage HCC (Yim et al., 2015). Therefore, there is an urgent need to develop effective and safe treatments for patients with HCC. HCC is accompanied by the dysregulation of various proteins. For example, the oncoprotein gankyrin, which is a component of the 19S regulatory cap of the proteasome and is an anti-apoptotic factor, is over-expressed in some tumor cell types such as HCC, esophageal squamous cell carcinoma, breast carcinoma, and endometrial carcinoma (Higashitsuji et al., 2000, 2005; Zhang J. et al., 2013). Gankyrin is mainly involved in protein–protein interactions, by binding to the ubiquitin ligase MDM2 to increase ubiquitination of p53 (Higashitsuji et al., 2005). Gankyrin can also enhance the phosphorylation of pRb, a tumor suppressor protein, by interacting with cyclin-dependent kinase 4 (CDK4) (Chattopadhyay et al., 2016). Overexpression of gankyrin reduces p53 and pRb expression, which contributes to oncogenic cell function and fate (Chapman et al., 2014; Chapman and McNaughton, 2015). Regulating the expression or activity of gankyrin is considered a therapeutic strategy for some cancers (Jiang et al., 2013; Sakurai et al., 2017). Gankyrin can be inhibited by C/EBPβ-histone deacetylase I complexes (Jiang et al., 2013), indicating that C/EBPs might provide critical signals for the regulation of gankyrin expression.

The C/EBP family supports the quiescent stage of the liver cells. One member of this family, C/EBPα, is an important transcription factor that participates in cell cycle regulation, cellular differentiation, and energy metabolism in various tissues (Jin et al., 2015; Lu G.D. et al., 2015). It has recently been reported that C/EBPα is upregulated in HCC cell lines, and results in hepatic lipid metabolic disturbance and further promotes HCC development (Lu et al., 2010; Liu et al., 2012; Huan et al., 2016; Cast et al., 2017). However, to date, there has been no investigation of the interaction between C/EBPα and gankyrin. This issue is examined in the present study in HCC cells.

Lipid metabolism in the liver is supported by PPARα, a member of the peroxisome proliferation activated receptor (PPAR) family (Bajaj et al., 2007). PPARα is a transcription factor and is expressed in various tissues, with high levels of expression in liver, intestine, heart, and kidney (Lee et al., 1995). Activation of PPARα occurs primarily through binding of ligands such as endogenous arachidonic acid, polyunsaturated fatty acids, and fatty acid-derived compounds (Fritsche, 2006; Georgiadi and Kersten, 2012; Rivera et al., 2014). Activated PPARα promotes uptake, utilization, and catabolism of fatty acids. PPARα upregulates genes involved in fatty acid transport, fatty acid binding and activation, and in peroxisomal and mitochondrial fatty acid β-oxidation. These effects are mediated through the formation of heterodimers with members of the retinoid X receptor (RXR) family of steroid receptors and by binding to specific DNA motifs termed PPAR-response elements (PPREs) (Li and Glass, 2004; Balanarasimha et al., 2014; Kersten, 2014). In addition to its role in regulating fatty acid metabolism, PPARα is involved in the initiation and development of cancers, such as human prostate cancer, breast cancer, colorectal cancer, and urinary bladder cancer (Gupta et al., 2000; Segawa et al., 2002; Yoshimura et al., 2003; Crowe and Chandraratna, 2004). PPARα is also involved in the growth and proliferation of liver cancer cell lines (Jeng et al., 2018).

The level of the protein gankyrin was found to be increased in 34 HCCs (Higashitsuji et al., 2000). It has also been found that the mechanism for ubiquitin proteasome system (UPS)-mediated elimination of C/EBPα during carcinogenesis required elevated levels of gankyrin, which interacts with an isoform of C/EBPα (Wang et al., 2010). However, the role and relationship of PPARα, C/EBPα, and gankyrin in the activity of arctigenin against HCC are obscure.

To elucidate the role and mechanism of action of arctigenin in HCC, we examined the expression of PPARα, C/EBPα, and gankyrin as well as the relationship between C/EBPα and gankyrin. This study demonstrates that arctigenin can inhibit HCC development and suggests a potentially new HCC therapeutic pathway.

## Materials and Methods

### Ethics Statement

This study was carried out in accordance with the Chinese recommendations and legislation on laboratory animals use and care. The protocol was approved by the Animal Ethics Committee of Lunan Pharmaceutical Group Co., Ltd. (Linyi, China).

### Cell Lines, Antibodies, and Reagents

Hep-3B (ATCC HB-8064) and HepG2 (ATCC HB-8065) cell lines were purchased from the American Type Culture Collection (ATCC, Manassas, VA, United States). Anti-gankyrin mAb (ab182576), anti-C/EBPα mAb (ab40764), and anti-PPARα mAb (ab8934) were obtained from Abcam (Abcam, Cambridge, MA, United States). Horseradish peroxidase (HRP)-conjugated goat anti-rabbit immunoglobulin G (IgG) was obtained from Santa Cruz (Santa Cruz Biotechnology, Santa Cruz, CA, United States). C/EBPα proteins were obtained from ORIGENE (Rockville, MD, United States), and PPARα was obtained from Creative Biomart (Shirley, NY, United States).

### Cell Proliferation Assay

The Cell Titer 96 Aqueous Non-Radioactive Cell Proliferation Assay was used to determine the number of viable proliferating cells according to the manufacturer’s instructions. Briefly, HepG2 and Hep3B cells were cultured in 96-well plates at a density of 5 × 10^3^ cells per well. Different concentrations of arctigenin were added to the wells and the cells were incubated for 24, 48, or 72 h at 37°C. Twenty microliters of 3-(4,5-dimethylthiazol-2-yl)-5-(3-carboxymethoxyphenyl)-2-(4-sulfophenyl)-2H-tetrazolium/phenazine methosulfate (MTS/PMS) solution was added to the 100 μl cell culture medium in each well. After incubation for 4 h, absorbance at 490 nm was measured using a Thermo MultiGo spectrophotometer (Thermo Fisher Scientific Inc., Milford, MA, United States).

### Colony Formation Assay

For the colony formation assay, 2 × 10^5^ cells HepG2 or Hep3B cells were cultured in 6-well plates for 24 h. Different concentrations of arctigenin were used to treat cells for 48 h. The cells were trypsinized and counted. Then, 200 cells per well were seeded into 96-well plates. Ten days later, the cells were fixed with 70% ethanol and stained with 0.5% crystal violet for 30 min; visible colonies were manually counted.

### Transwell Migration/Invasion Assay

To measure cell migration, transwell inserts with 8-μm pore sizes were placed in 24-well plates. HepG2 and Hep-3B cells were treated with 20 μM arctigenin for 2 days. Then, 1 × 10^5^ HepG2 and Hep-3B cells were seeded in the upper chamber. The lower chamber was filled with Dulbecco’s Modified Eagle’s Medium (DMEM) containing 10% fetal bovine serum (FBS). Twenty-four hours later, cells migrating to the underside of the transwell membrane were fixed with 4% paraformaldehyde and stained with crystal violet. The migratory cells were viewed with a NIKON microscope and their numbers were counted in five different fields of view (Han et al., 2017). Matrigel-coated transwell chambers (BD Biosciences, United States) were used to detect cell invasion. HepG2 and Hep3B cells were seeded on the matrigel matrix in the upper chamber. DMEM culture medium was added to the bottom chamber with 10 nM of arctigenin. After 48 h, the cells that had invaded the matrigel-coated membranes were fixed with paraformaldehyde and stained with crystal violet (Zhang et al., 2015).

### Western Blot Analysis

HepG2 and Hep-3B cells were treated with 20 μM arctigenin for 48 h. The cells were harvested and lysed using cell lysis buffer (25 mM Tris-HCl pH 7.6, 150 mM NaCl, 1% NP-40, 1% sodium deoxycholate, 0.1% SDS) (Thermo Fisher, Milford, MA, United States). Total protein was extracted and separated by 12% sodium dodecyl sulfate polyacrylamide gel electrophoresis (SDS-PAGE), then transferred to polyvinylidene fluoride (PVDF) membranes. The membranes were blocked with 5% non-fat milk for 1 h at 37°C, and then incubated with primary antibodies for 1 h at 37°C. Anti-gankyrin mAb was diluted at 1:1000, anti-C/EBPα mAb was diluted at 1:2000, and anti-PPARα mAb was diluted at 1:1000. After secondary antibody incubation for 1 h at room temperature (RT), bands were monitored using enhanced chemiluminescent (ECL) reagent.

### Real-Time Quantitative Reverse Transcription Polymerase Chain Reaction (QRT-PCR)

HepG2 and Hep-3B cells were treated with 20 μM arctigenin for 48 h. Total RNA was extracted from cells using TRIZOL reagent (Invitrogen, Grand Island, NY, United States). One microgram of each RNA sample was used to synthesize first strand cDNA using a ReverTra Ace qPCR RT Master Mix kit (TOYOBO Co., Ltd., Osaka, Japan) according to the manufacturer’s instructions. Real-time PCR detection with SYBR Green master reagent (TOYOBO Co., Ltd., Osaka, Japan) was performed with an ABI 7300 instrument (Applied Biosystems, Foster City, CA, United States). Expression of gankyrin, C/EBPα, and PPARα in patients’ tumor tissues was detected by QRT-PCR. The following primer sequences were used:

Gankyrin P1, 5′-GCCAAGGGTAACTTGAAGATGA-3′ and

Gankyrin P2, 5′-TCACAGGCTAAGTGTAGAGGAG-3′ (Yang et al., 2016);

C/EBPα P1, 5′-AAGAAGTCGGTGGACAAGAACAG-3′ and

C/EBPα P2, 5′-GCGGTCATTGTCACTGGTCA-3′ (Paz-Priel et al., 2011);

PPARα P1, 5′-CTACGAGGCCTACCTGAAGAACTT-3′ and

PPARα P2, 5′-CGAGCGTCTTCTCAGCCATAC-3′ (Liu et al., 2016);

GAPDH P1, 5′-GAGTCAACGGATTTGGTCGT-3′ and

GAPDH P2, 5′-AATGAAGGGGTCATTGATGG-3′.

### Plasmid Construction

Different human gankyrin promoter regions (containing positions -750 to +20, -500 to +20, -450 to +20, -400 to +20, -350 to +20, -300 to +20, and -250 to +20) were cloned into a *Xho*I/*Hin*dIII digested pGL-3 vector. The primers are listed in **Table 1**. *Xho*I was introduced in all the forward primers, while *Hin*dIII was introduced to reverse primers. All the recombination plasmids were verified by DNA sequence analysis. Gankyrin promoter DNA, from -450 to -401, was synthesized by Shanghai Shenggong, Co.

**Table 1:** Primers for construction.

Primer	Sequence (5′ to 3′)
Forward primer	
p-750	ATCTCGAGTAGATATCAGGTGGCTCCCA
p-500	ATCTCGAGCTTCCGCCTGCCACTGCAGG
p-450	TACTCGAGGGGGACAGGACCTTTAGGCT
p-400	ATCTCGAGGCTGTCATCCTGGGCCAATT
p-350	TTCTCGAGTTCACACGGTTCCACTCAAA
p-300	ATCTCGAGTTTCATATGGCCGACTCAAC
p-250	TACTCGAGCGCTAGGATCCCTGGGACTT
Reverse primer	
p+20	CGAAGCTT TTAGACACAGAGCCGTCCAT
C/EBPα	
pF	AATACTCGAGATGGAGTCGGCCGACTTCTACG
pR	ATATAAGCTTTCACGCGCAGTTGCCCATGGCC
PPARα	
pF	ATATGGGCCCATGGTGGACACGGAAAGCCCAC
pR	CGGCGGATCCTTAATACATGTCCCTGTAGATC

*C/EBPα* and *PPARα* DNA fragments were subcloned into a pcDNA3.1(-) vector. *Xho*I and *Hin*dIII were introduced into *C/EBPα*-specific primers. *Apa*I and *Bam*HI were introduced into *PPARα*-specific primers (**Table 1**).

### Transient Transfection and Luciferase Assays

HepG2 cells were used for luciferase assays. Recombinant pGL-3 plasmids containing different regions of the gankyrin promoter were transiently transfected into HepG2 cells. After 48 h, transfected HepG2 cells were treated with 5 nM arctigenin for 16 h. Three replicate experiments were performed for each treatment. The cells were lysed, and luciferase activity was quantified using the Dual-Luciferase Reporter Assay System (Promega, Madison, WI, United States) according to the manufacturer’s instructions. To evaluate whether C/EBPα and PPARα could bind to the gankyrin promoter, pcDNA3.1-C/EBPα/PPARα or pGL3-Gan-P50 (-450 to -401) were transfected into HepG2 cells, which were then treated with 10 nM arctigenin. Luciferase activity was detected using the luciferase assay system (Promega, Madison, WI, United States).

### Co-immunoprecipitation (Co-IP) Assay

A co-immunoprecipitation (Co-IP) assay was performed to determine the relationship between C/EBPα/PPARα and the gankyrin promoter. The Co-IP kit was used according to the manufacturer’s protocol (Li J. et al., 2015). Nuclear extracts of HepG2 cells and liver tissues were immunoprecipitated with anti-C/EBPα or anti-PPARα antibodies. The resulting samples were analyzed using the immunoblotting procedures described above (Li J. et al., 2015).

### Chromatin Immunoprecipitation (ChIP) Assay

A ChIP assay was conducted using a ChIP kit (Epigentek Group, Inc., Brooklyn, NY, United States) and the method of Li J. et al. (2015). Briefly, HepG2 cell DNA was sheared to 200–1,000 base pair lengths. The chromatin solution was then immunoprecipitated with 10 μg anti-C/EPBα antibodies, anti-PPARα antibody, or non-immune rabbit IgG as a negative control. The number of cycles to amplify PCR products was within the linear range; the PCR products were resolved on a 2% agarose gel. The following primers were used:

P1, 5′-TTGTC**CC**TTCCGCCTGCCAC-3′ and

P2, 5′-TGGAGAATGGAGTCAGGTAC-3′.

### Binding of Arctigenin With C/EPBα or PPARα

The hollow fiber-based ligand fishing (HFLF) strategy was used to determine whether arctigenin could bind to C/EPBα or PPARα. Hollow fiber membranes with an inner diameter of 500 μm and a pore size of 0.2 μm were purchased from GE Healthcare. Briefly, C/EBPα and PPARα proteins were injected into the lumen of hollow fibers that had been treated with acetone, methanol, and ddH_2_O. The hollow fiber membranes were filled with proteins and sealed by heating; they were then placed into an arctigenin extract (10 mg/ml), and incubated in an ultrasonic bath at 37°C for 30 min. After washing with phosphate-buffered saline (PBS), bound compounds formed in the lumen were dissociated with 30 μl of methanol. The fluid was centrifuged at 10,000 × *g* for 10 min. Twenty microliters of supernatant was used for liquid chromatography–mass spectrometry (LC–MS) analysis. The flow chart is shown in **Figure 4A**.

### Animal Experiment

Female BALB/c nude mice (6–8 weeks old) received a subcutaneous injection of HepG2 cells (1 × 10^6^ cells per mouse) into the left flank on day 1. On day 2, the mice were treated with arctigenin (1 mg/kg or 10 mg/kg) by subcutaneous injection. Tumor volumes were measured every 3 days, and the survival rate was calculated. The data are presented as mean volume ± SE.

### Immunohistochemistry

Immunohistochemical (IHC) staining of formalin-fixed tumor tissues was performed to detect C/EBPα, PPARα, and gankyrin levels. Paraffin embedded tissue sections were de-paraffinized and re-hydrated. Antigen recovery was performed by boiling in trisodium citrate buffer (10 mM, pH 6.0). Then, sections were immunostained with antibodies against C/EBPα, PPARα, and gankyrin for 2 h at RT, and 1 h at RT with HRP-labeled IgG. 3,3-diaminobezidine (DAB) staining was used to evaluate protein levels.

### Statistical Analyses

Results are expressed as mean ± standard deviation (SD). Tumor volumes are expressed as mean ± standard errors (SE). Statistical analyses were performed using Student’s *t*-test using GraphPad prism 5.0, with *P* < 0.05 considered to be statistically significant.

## Results

### Arctigenin Inhibits Proliferation, Migration, and Colony Formation in HepG2 but Not Hep3B Cells

**FIGURE 1:**
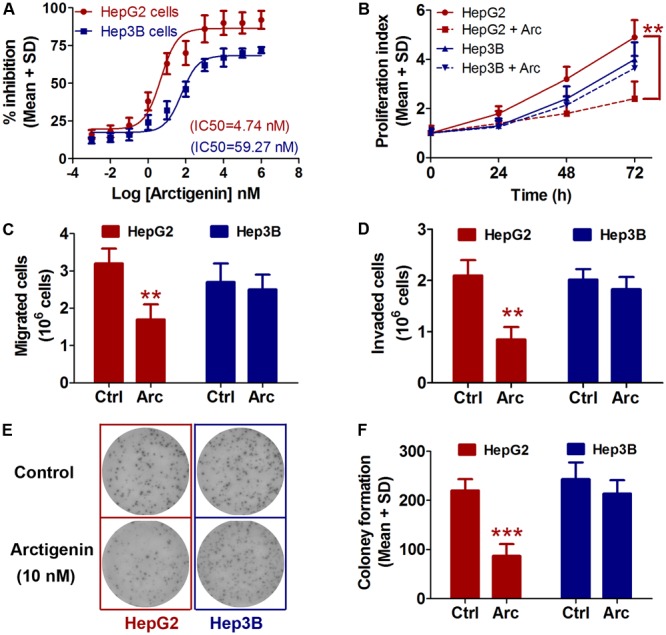
Arctigenin inhibits proliferation, migration, and colony formation in HepG2 but not Hep3B cells. **(A)** IC_50_ values of arctigenin on HepG2 and Hep3B cells determined using a cell proliferation assay. **(B)** Proliferation of arctigenin-treated HepG2 and Hep3B cells at 0, 24, 48, and 72 h. Experiments were performed in triplicate for each group. **(C,D)** Migration and invasion of HepG2 and Hep3B cells treated with arctigenin. **(E)** Representative micrographs and **(F)** quantification of crystal violet-stained cell colonies. The control cells were treated with dimethyl sulfoxide. ^∗∗^*P* < 0.01, ^∗∗∗^*P* < 0.001, arctigenin-treated group vs. the control group. Arc, arctigenin; Ctrl, control.

The effects of treatment for 24 h with a range of arctigenin concentrations (10^-3^–10^6^ nM) on HepG2 and Hep3B liver cancer cell lines cells were determined. As shown in **Figure 1A**, arctigenin had a different anti-proliferative effect in the two cell types: IC_50_ (maximal inhibitory concentration) was 4.74 nM in HepG2 cells, but 59.27 nM in Hep3B cells. Assessment of cell proliferation rates after treatment with 10 nM arctigenin showed that HepG2 cells were more sensitive than Hep3B cells (**Figure 1B**). Analyses of the effects of arctigenin on migration, invasion, and colony formation showed that the rates of the three parameters were reduced by 47.1, 59.6, and 60.5%, respectively, in HepG2 cells (**Figures 1C–F**). However, there were no differences in these parameters when Hep3B cells were treated by arctigenin. Taken together, these results indicate that arctigenin was able to inhibit proliferation and migration of HepG2 cells, but not Hep3B cells, suggesting that arctigenin may act in different pathways and have different anti-tumor effects in the two cell lines.

### Arctigenin Inhibits the Expression of Gankyrin in HepG2 but Not Hep3B Cells

**FIGURE 2:**
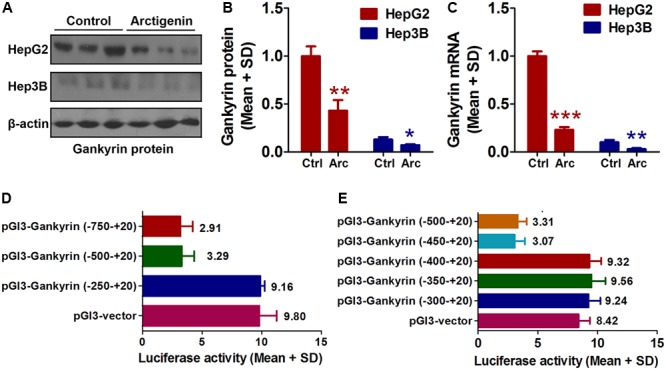
Arctigenin inhibits expression of gankyrin in HepG2 but not Hep3B cells. **(A,B)** Western blot showing level of gankyrin protein in HepG2 and Hep3B cells with or without arctigenin treatment. **(C)** Gankyrin mRNA levels in HepG2 and Hep3B cells with or without arctigenin treatment. **(D)** Human gankyrin promoter-reporter constructs (from –750 to +20 truncated into different lengths) were transfected into HepG2 cells following arctigenin treatment. The activity of the gankyrin promoter (from –500 to –250) was blocked by arctigenin. **(E)** Human gankyrin promoter-reporter constructs (from –500 to +20 truncated into different lengths) were transfected into HepG2 cells following arctigenin treatment. The activity of the gankyrin promoter (from –450 to –400) was blocked by arctigenin treatment. Basal activity levels measured in cells transfected with the pGL-3 empty vector were set as 1. Triplicate experiments were conducted for each set. ^∗^*P* < 0.05, ^∗∗^*P* < 0.01, ^∗∗∗^*P* < 0.001.

As mentioned earlier, the levels of gankyrin are increased in many cancers and, therefore, gankyrin is considered a potential therapeutic target in liver cancer (Nanaware et al., 2014; Zamani et al., 2017). To investigate whether gankyrin is involved in the potential anti-tumor activity of arctigenin, we investigated the levels of gankyrin protein in HepG2 and Hep3B cells by immunoblotting and examined gene expression by QRT-PCR. The results showed comparatively high protein and gene expression levels in HepG2 cells but only a basal level of both in Hep3B cells. Arctigenin treatment significantly reduced gankyrin mRNA and protein levels in HepG2 cells (77 and 56% reduction in levels, respectively, **Figures 2A–C**). To investigate the mechanism of this inhibitory effect, different segments of the gankyrin promoter were cloned and inserted into a pGL-3-luciferase vector, and then transfected into HepG2 cells. After arctigenin stimulation, luciferase activity was assayed (**Figure 2D**). The analysis showed that luciferase activity was not influenced when the promoter region -250 to +20 was inserted into the pGL-3-luciferase vector compared with the control group (pGL3-vector). However, for the region from -250 to -500 [pGL3-*gankyrin*-(-500-+20)], luciferase activity was significantly decreased after arctigenin treatment; no further reduction was observed if the region was extended from -750 to +20 (**Figure 2D**). These results indicate that the potential target of arctigenin on the gankyrin promoter was from -500 to -250. Subsequently, this region of the promoter was further cut into smaller lengths, and luciferase assays demonstrated that the domain from -450 to -400 was the main active site for arctigenin binding (**Figure 2E**).

### C/EBPα and PPARα Are Involved in Gankyrin Expression, Which Is Influenced by Arctigenin Treatment

**FIGURE 3:**
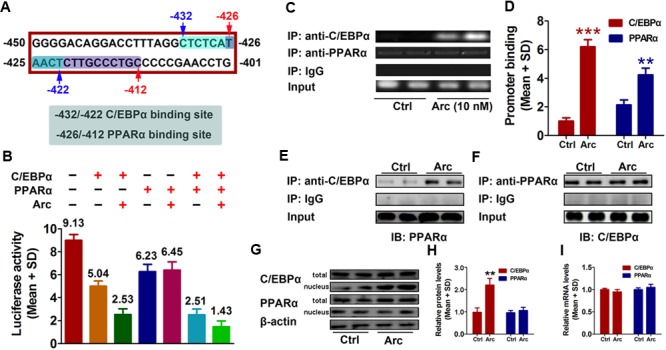
C/EBPα and PPARα are involved in gankyrin expression, which is modulated by arctigenin. **(A)** The predicted target sequences of human C/EBPα and PPARα located on the gankyrin promoter (–450 to –401). **(B)** C/EBPα and PPARα show a synergistic inhibitory activity on a gankyrin-reporter plasmid. **(C,D)** Binding of C/EBPα and PPARα to the gankyrin promoter in a ChIP assay **(C)** and calculated **(D)**. **(E,F)** Binding of C/EBPα to PPARα in a Co-IP assay, using anti-C/EBPα and anti-PPARα antibodies for immunoprecipitation. **(G,H)** Total and nuclear protein levels of C/EBPα, and PPARα in HepG2 cells with or without arctigenin treatment as determined by immunoblotting **(G)** and calculated **(H)**. **(I)** *C/EBPα* and *PPARα* mRNA levels in HepG2 cells with or without arctigenin treatment were determined by QRT-PCR. Triplicate experiments were conducted for each set. ^∗∗^*P* < 0.01.

To further investigate potential factor(s) involved in arctigenin-mediated inhibition of gankyrin, we used the online software JASPAR^1^ and TFBIND^2^ to profile the -450 to -401 region of the *gankyrin* promoter. We identified two overlapping sequences for the binding sites of C/EBPα (-432 to -422) and PPARα (-426/-412) (**Figure 3A**). To determine whether C/EBPα and/or PPARα mediated arctigenin*-*induced inhibitory effects, we constructed expression plasmids for C/EBPα and PPARα. pcDNA3.1(-)-C*/*EBPα and/or pcDNA3.1(-)-PPARα plasmid were co-transfected with pGL3-gankyrin-(-450/-401) into HepG2 cells. A luciferase activity assay demonstrated that C/EBPα and PPARα could individually inhibit luciferase activity, and that this inhibitory effect was enhanced when both were present. Arctigenin significantly decreased luciferase activity in combination with C/EBPα, but not with PPARα (**Figure 3B**). A ChIP assay to investigate the binding status of C/EBPα and PPARα on the gankyrin promoter (-450 to -401) showed that arctigenin treatment significantly increased C/EBPα binding but did not affect binding of PPARα in this promoter region (**Figures 3C,D**). Although the predicted binding sites of C/EBPα and PPARα in the gankyrin promoter (from -450 to -401) overlap, the luciferase assay demonstrated that there was no competition between the transcription factors, rather that they showed synergism in their activities. These results implied that C/EBPα and PPARα interacted, and that this interaction was modified by arctigenin to inhibit gankyrin expression. To verify this interpretation, a Co-IP assay was used to detect whether protein–protein binding occurred between C/EBPα and PPARα and whether it was influenced by arctigenin. The analysis showed that C/EBPα could bind with PPARα. An oversaturated C/EBPα antibody was used to immunoprecipitate C/EBPα, and arctigenin significantly increased PPARα binding to C/EBPα (**Figure 3E**), and in accordance with IP-PPARα (**Figure 3F**). Immunoblot and QRT-PCR assays demonstrated that the levels of C/EBPα and PPARα mRNA and protein did not differ with arctigenin treatment. Compared with the control group, arctigenin treatment significantly increased the movement of C/EBPα from the cytoplasm to the nucleus (**Figures 3G–I**), which implies that C/EBPα, not PPARα, mediated the arctigenin*-*triggered inhibitory effects on gankyrin expression.

### Arctigenin Binds With C/EBPα *in Vitro*, but Not PPARα

**FIGURE 4:**
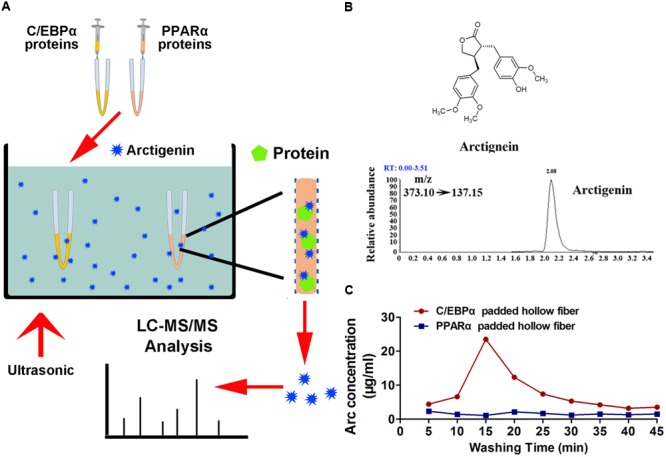
Arctigenin binds with C/EBPα but not PPARα *in vitro.* **(A)** Schematic illustration of an HFLF assay combined with LC–MS. **(B)** Structure and representative extracted ion chromatograms of spiked arctigenin (multiple reaction monitoring model). **(C)** Arctigenin concentration vs. washing time profiles in an HFLF assay. Triplicate experiments were conducted for each set.

The potential mechanisms by which C/EBPα and PPARα might mediate arctigenin-triggered inhibition of gankyrin were next investigated using a hollow fiber-based ligand fishing (HFLF) strategy to determine whether C/EBPα and/or PPARα bind with arctigenin. Microporous hollow fibers filled with C/EBPα or PPARα were used as baits to hook arctigenin, followed by dissociation of target-ligand complexes. The identity of the ligand (arctigenin) was then confirmed by LC–MS. The screening procedure using HFLF and HPLC–MS/MS is shown in **Figure 4A**. Representative extracted ion chromatograms after spiking with arctigenin (multiple reaction monitoring model) are shown in **Figure 4B**. Different washing times, ranging from 5 to 45 min, after incubation were studied under ultrasonic conditions using PBS buffer (pH 7.4). As shown in **Figure 4C**, in the C/EBPα-baiting group, the concentration of arctigenin increased after a 5-min wash, peaked at 15 min, and reached a plateau at 30 min. However, the concentration of arctigenin was unchanged in the PPARα-baiting group. The results indicated that arctigenin could bind with C/EBPα, but not PPARα.

### Gankyrin Expression Is Increased and Is Negatively Correlated With C/EBPα and PPARα in Liver Cancer Tissues

**FIGURE 5:**
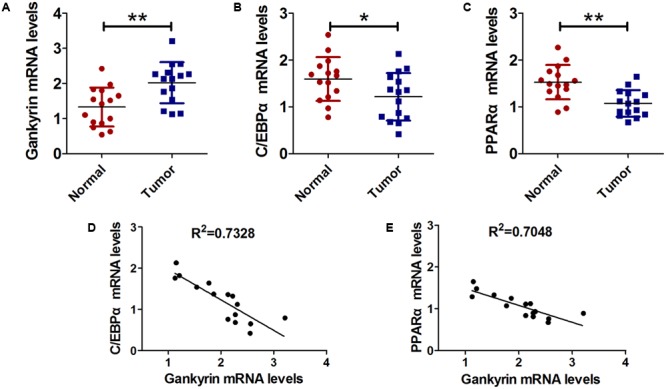
Gankyrin expression was increased and negatively correlated with *C/EBPα* and *PPARα* levels in liver cancer tissues. **(A–C)** QRT-PCR analysis was performed to detect the expression of *C/EBPα* **(A)**, *PPARα* **(B)**, and gankyrin **(C)** in liver cancer and cancer-adjacent normal tissues. *n* = 15, ^∗^*P* < 0.05, ^∗∗^*P* < 0.01, cancer tissue vs. adjacent normal tissue. **(D)** Association between gankyrin levels and *C/EBPα* expression levels. **(E)** Association between gankyrin levels and *PPARα* expression levels.

Next, we examined the relationship of C/EBPα, PPARα, and gankyrin expression in liver cancer tissues using QRT-PCR. We found that the level of gankyrin mRNA was greater in liver cancer tissues than normal tissues (**Figure 5A**). In contrast, the *C/EBPα* and *PPARα* mRNA levels were both lower in liver cancer tissues compared to normal tissues (**Figures 5B,C**). We used GraphPad Prism 5 software to investigate the associations between C/EBPα, PPARα, and gankyrin expression. A regression analysis showed that gankyrin mRNA levels were negatively correlated with the levels of *C/EBPα* (*R*^2^ = 0.7328, **Figure 5D**) and *PPARα* mRNA (*R*^2^ = 0.7048, **Figure 5E**).

### Arctigenin Inhibits Tumorigenesis in Liver Cancer by Inhibiting *Gankyrin* Expression via C/EBPα and PPARα

**FIGURE 6:**
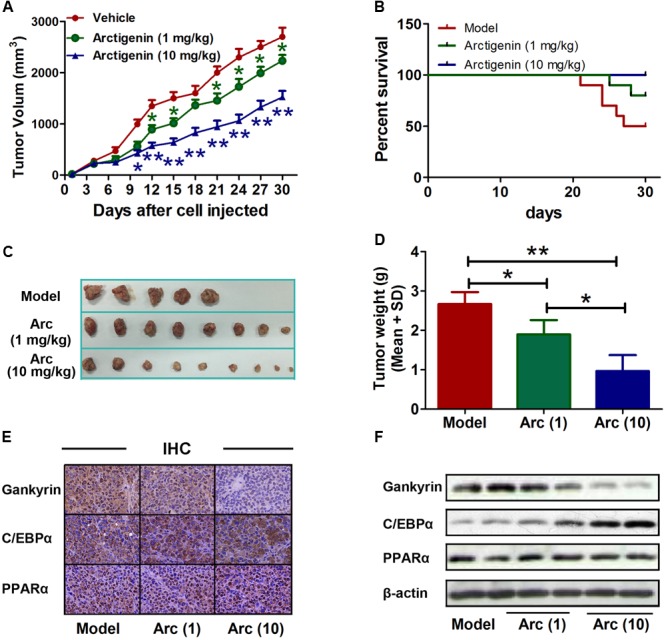
Arctigenin inhibits liver cancer tumorigenesis *in vivo.* **(A)** Volumes of HepG2 tumors *in vivo* were measured every 3 days with or without arctigenin treatment. *n* = 10, ^∗^*P* < 0.05, ^∗∗^*P* < 0.01. **(B)** Representative survival curves of each group. **(C,D)** Representative tumors and weights in each group. *n* = 10, ^∗^*P* < 0.05. **(E)** Representative images of IHC assay of tumor tissues with gankyrin, C/EBPα, and PPARα. **(F)** Nucleus proteins were extracted from tumor tissues of each group, and the levels of gankyrin, C/EBPα, and PPARα were detected by immunoblotting.

The role of arctigenin on tumorigenesis *in vivo* was investigated in BALB/c nude mice that had received a subcutaneous injection of HepG2 cells with or without arctigenin treatment (1 mg/kg or 10 mg/kg). Tumor volumes were measured every 3 days. We found that arctigenin decreased HepG2 cell growth *in vivo* (**Figures 6A,C,D**). There were no deaths in the control (untreated) group of mice throughout the period of the experiment (30 days). However, a 50% (*n* = 5) mortality rate occurred in the positive control group injected with HepG2 cells; all deaths occurred toward the end of the experimental period (days 21–27) (**Figure 6B**). In contrast, in the mice treated with arctigenin, the mortality rate at both 1 mg/kg (20%) and 10 mg/kg (no death) were reduced compared to the positive control; the deaths occurred toward the end of the experimental period (days 25 and 28). The possible roles of C/EBPα, PPARα and gankyrin in the protective effect of arctigenin in the HepG2 cell-xenografted mice were investigated by IHC staining in tumor tissues of each group of mice. As shown in **Figure 6E**, gankyrin staining decreased with the dose of arctigenin; C/EBPα nuclear levels increased with dose of arctigenin; PPARα levels did not vary between the three groups. To confirm this outcome, nuclear proteins were extracted from the tumor tissues of each group quantified using immunoblots (**Figure 6F**). The results were consistent with those of IHC staining.

## Discussion

Arctigenin is an active natural ingredient present in the seeds of *Arctium lappa*. The antitumor activity of this lignan has been studied widely. An oral drug, GBS-01, which contains high levels of arctigenin was found to have a beneficial effect on pancreatic cancer in a phase I clinical study (Ikeda et al., 2016). Arctigenin selectively induced apoptosis by upregulating apoptosis-related genes, including *Bax, Fas*, and caspase in A549 cells (Susanti et al., 2013). In the human pancreatic cancer cell line PANC-1, arctigenin inhibited growth by suppressing the unfolded protein response (UPR), which is involved in protection against cell death (Sun et al., 2011). Arctigenin has also been shown to suppress heat shock response in tumor cells (Ishihara et al., 2006). The anti-tumor mechanisms of arctigenin vary among tumor types. However, its mode of action is still unclear in some cancers, such as HCC.

In the present study, we showed that arctigenin efficiently inhibited HCC growth, invasion, and metastasis *in vitro*. Arctigenin also decreased the levels of gankyrin in HepG2 and Hep3B cell lines. As gankyrin is overexpressed in human liver cancers (Higashitsuji et al., 2000), we evaluated the levels in two liver cancer cell lines and found high levels in HepG2 cells, but low levels in Hep3B cells. In our previous study, arctigenin was found to inhibit the growth of HepG2 cells, but to have no effect on Hep3B cell growth (data unpublished). This suggests that gankyrin might influence the activity of arctigenin in HCCs. Gankyrin can increase tumor cell growth and promote invasiveness and metastasis by activating the PI3K/AKT pathway (Fu et al., 2011). Our results showed that the levels of gankyrin were higher in tumor tissues than in adjacent tissues. However, when HepG2 cells were treated with arctigenin, the level of gankyrin markedly declined. Moreover, a luciferase reporter assay further indicated that arctigenin reduced *gankyrin* promoter activity. Two regulatory factors, C/EBPα and PPARα, which have binding sites at -432 to -422 and -426 to -412, respectively, in the gankyrin promoter sequence, play a critical role in regulating gankyrin expression. The region of the gankyrin promoter from -450 to -400 was critical for the effect of arctigenin on C/EBPα- and PPARα-modulated gankyrin expression.

Our binding screening assay suggested that arctigenin could bind to PPARα, but not to C/EBPα. When bound to PPARα, arctigenin did not affect the interaction between C/EBPα and PPARα in a Co-IP detection. However, the binding of arctigenin and PPARα disturbed PPARα binding to the gankyrin promoter and further reduced gene transcription.

In this study, we showed that arctigenin inhibits HCC growth by reducing gankyrin expression. It was previously reported that gankyrin could promoted HCC growth through the PI3K/AKT pathway (Fu et al., 2011). Gankyrin also sustains PI3K signal activation and promotes colorectal cancer aggressiveness and progression (He et al., 2016). Arctigenin can induce specific cytotoxicity in HCC by deactivating the PI3K/AKT pathway (Lu Z. et al., 2015). Although we did not examine PI3K/AKT pathway-related proteins, our results are consistent with those from previous studies. The present investigation identified the molecular mechanism for arctigenin activity against HCC, and suggested a new therapeutic strategy for HCC.

## Author Contributions

JL and J-cY contributed to the conception and design of the study and approved the final version to be submitted. YS, Y-jT, and C-hS performed the experiments. L-lZ and TL collected samples from experimental animals. G-mZ and ZL performed the cell experiments. Z-zL and B-bL performed the sample detection. JL and YS contributed to drafting the article and data analysis and interpretation.

## Conflict of Interest Statement

YS, Y-jT, Z-zL, B-bL, C-hS, TL, L-lZ, ZL, G-mZ, J-cY, and JL were employed by Lunan Pharmaceutical Group Co., Ltd. The reviewer LV and handling Editor declared their shared affiliation.

## Footnotes

1 ^1^http://jaspar.genereg.net/cgi-bin/jaspar_db.pl

2 ^2^http://tfbind.hgc.jp/

## Abbreviations

C/EBPα: CCAAT/enhancer binding protein alpha
PPARα: peroxisome proliferator-activated receptor alpha
QRT-PCR: real-time quantitative RT-PCR
